# Securing structured transitional care for adolescents with type 1 diabetes: a qualitative study of implementation barriers and facilitators prior to implementation– the STEPSTONES-Implement project

**DOI:** 10.1186/s12913-026-14576-1

**Published:** 2026-04-17

**Authors:** Anna Lena Brorsson, Carina Sparud-Lundin, Markus Saarijärvi

**Affiliations:** 1 Department of Women’s and Children’s Health, Karolinska Institutet, Stockholm, Sweden; 2 Astrid Lindgren Children’s Hospital, Karolinska University Hospital, Stockholm, Sweden; 3 University of Gothenburg Institute of Health and Care Sciences, Gothenburg, Sweden; 4 University of Gothenburg Centre for Person-Centered Care, Gothenburg, Sweden; 5 Department of Cardiology, Danderyd University Hospital, Stockholm, Sweden; 6 Karolinska Institute Department of Neurobiology Care Sciences and Society, Huddinge, Sweden

**Keywords:** Adolescents, Implementation science, Person-centered care, Qualitative research, Transition to adult care, Type 1 diabetes

## Abstract

**Background:**

Adolescents and young adults with type 1 diabetes (T1D) face challenges during the transition from pediatric to adult healthcare, including gaps in care and deteriorating health outcomes. Although structured transition programs have demonstrated effectiveness, few components of transitional care are implemented in practice. Swedish Transition Effects Project Supporting Teenagers with chrONic mEdical conditions (STEPSTONES) is a person-centered, multicomponent intervention developed to support adolescents with chronic conditions during this transition. Prior to implementation in pediatric diabetes care, there is a need to understand the implementation context that might influence program adoption. The aim of this study was to explore healthcare professionals’ perspectives on barriers to and facilitators for implementing the STEPSTONES transition program for adolescents with T1D in two pediatric diabetes outpatient settings in Sweden prior to implementation.

**Methods:**

Qualitative inductive design was employed. Data was collected during 2024 – 2025 through three focus group interviews with registered nurses (*n* = 8), physicians (*n* = 3) and individual interviews with healthcare managers (*n* = 2) and the chair for a national patient organization for youth with T1D (*n* = 1). Participants were recruited from two pediatric diabetes outpatient clinics at a university hospital in Sweden prior to program implementation. Data was analyzed using thematic analysis.

**Results:**

An overarching theme, *Conceptualizations of structured transitional care*, was identified, encompassing three themes: *openness to changes in the transition care processes, ensuring dedicated responsibility for transitional care and supporting a youth-oriented approach.* Key facilitators included a strong perceived need for structured transition support, alignment of the program with existing practices, leadership engagement, and the appointment of a dedicated transition coordinator. Barriers were related primarily to contextual constraints, such as limited resources, challenges in collaboration between pediatric and adult care, and healthcare professionals’ uncertainty regarding how to discuss sensitive topics and psychosocial support. healthcare professionals’ uncertainty regarding

**Conclusions:**

These findings indicate favorable preconditions for implementing the STEPSTONES transition program in pediatric diabetes care. However, successful implementation will depend on addressing cross-organizational contextual barriers, strengthening recipients’ competence in adolescent-centered care, and investing in active and sustained facilitation. In accordance with the Integrated Promoting Action on Research Implementation in Health Services (i-PARIHS) framework that we used as a post hoc interpretative framework, the findings highlight that implementation success requires alignment between the innovation, recipients, and context, with facilitation playing a pivotal role. These insights may inform the implementation of structured transition programs for adolescents with chronic conditions in routine healthcare.

**Supplementary information:**

The online version contains supplementary material available at 10.1186/s12913-026-14576-1.

## Introduction

In Sweden, type 1 diabetes (T1D) is one of the most common chronic conditions, with approximately 8700 children and adolescents (0–18 years) living with this life-long condition [1]. Maintaining optimal glucose management is essential to minimize the risk of both short- and long-term complications [2]. Adolescence represents a critical developmental phase marked by the pursuit of autonomy and substantial transformations across physical, cognitive, and emotional domains. For individuals with T1D, this period also necessitates a gradual transfer of responsibility for self-management from parents to adolescents [3].

Challenges in diabetes care are inadequate preparation for transfer to adult care and transition to adulthood, the risk of gaps in care between pediatric and adult care, sub-optimal glycemic management, and increased hospital admissions after transfer [3]. In general, glucose outcomes have improved in recent decades in several countries, probably related to national quality improvements, access to health care coverage and advanced diabetes technologies for insulin administration and continuous glucose measurement for people with T1D [4]. However, young people aged approximately 18–25 years have the worst glucose outcomes of all age groups, and there is a deterioration in disease management during these years, when young people are transferred from a pediatric to an adult setting [1, 4]. Research on youth with T1D indicates that transition interventions may be effective in maintaining glycated hemoglobin (HbA1c), reducing ketoacidosis episodes, improving diabetes management, and reducing diabetes-related distress after the transition to adult care [5, 6]. International guidelines recommend a planned and organized transition from pediatric to adult care [3, 7, 8]. Overall, these findings indicate the importance of a well-planned and coordinated transition and transfer of care from childhood to adult life and care. However, reported barriers to implementing recommended interventions include shortages of personnel, limited resources and funding, as well as a lack of specialized expertise in adolescent [9].

STEPSTONES (Swedish Transition Effects Project Supporting Teenagers with chrONic mEdical conditionS) is a research program aimed at developing, evaluating and implementing a person-centered transition program to support teenagers with chronic conditions in their transition to adulthood and transfer to adult care. The STEPSTONES transition program is a multicomponent intervention adapted from a Belgian study [10] and further developed using the Medical Research Council’s (MRC) framework for complex interventions [11] along with intervention mapping and an extensive needs assessment of the target population [12–15]. The intervention has been evaluated in a randomized controlled trial (RCT) for adolescents with congenital heart disease (STEPSTONES-CHD) [16] and is soon being completed in adolescents with T1D (STEPSTONES-DIAB) [17]. The results from the STEPSTONES-CHD study demonstrated statistically significant improvements in patient empowerment, which was the primary outcome. Additionally, secondary outcomes included reduced parental involvement, greater satisfaction with physical appearance, and enhanced disease-related knowledge [16]. A process evaluation indicated that the program was implemented with a high degree of fidelity. However, challenges were identified in the implementation of specific components of the transition program, particularly low fidelity to peer support and the joint transfer meetings with adult care [18]. Consequently, several factors affecting implementation were identified.

Despite growing evidence on the efficacy of transition programs across various chronic conditions, implementation in healthcare is poor [9, 19, 20]. There are few evaluations regarding the implementation of transition programs in clinical practice, and these point out the need to consider the unique role of organizational behavior and contextual factors in implementation [19]. We recently published a study exploring barriers and facilitators for the implementation of the STEPSTONES transition program in pediatric cardiology, and the findings highlighted the need for leadership support, contextual adaptation and uncertainties regarding nurses’ self-efficacy in providing psychosocial components of transitional care [21]. Considering these findings and the dire need for contextual understanding when complex interventions are implemented, we endeavor to explore whether and to what extent these findings are reflected or contrasted in the pediatric diabetes setting. As implementation contexts are dependent on both local, regional and disease-specific factors we want to endeavor to determine what similarities and differences are present [22]. Therefore, prior to implementation, we aimed to explore barriers and facilitators for implementing the STEPSTONES transition program for adolescents with T1D in two pediatric diabetes outpatient settings in Sweden.

## Method

### Aim

The aim of this study was to explore healthcare professionals’ perspectives on barriers to and facilitators for implementing the STEPSTONES transition program for adolescents with T1D in two pediatric diabetes outpatient settings in Sweden prior to implementation.

### Design

Qualitative inductive design was used, and the reporting of this study followed the Standards for Reporting Qualitative Studies (SRQR) guidelines [23].

### Setting

The study was conducted at two pediatric diabetes clinics at Astrid Lindgren Children’s Hospital, Karolinska University Hospital in Stockholm, Sweden, prior to the implementation of the STEPSTONES transition program. Together, these clinics provide care to 14% of children and adolescents with T1D in Sweden [1]. The two clinics transfer patients to approximately eight adult clinics that cover a larger metropolitan area. The medical follow-up of children and adolescents with T1D adheres to international guidelines [24] and is delivered by a multidisciplinary team with expertise in pediatric and diabetes management. Clinic visits are generally scheduled every three months but are individualized according to patient needs and resource availability, with consultations alternating between physicians and diabetes specialist nurses. There is currently no standardized protocol nationally or locally in T1D for preparing adolescents for transition to adult life and adult healthcare services. Usual care means that in the final year before transfer, healthcare professionals typically initiate transition discussions, primarily to identify the receiving adult diabetes clinic. At 18 years of age, a formal referral is made to the agreed-upon clinic. At one of the involved pediatric diabetes clinic, the final physician visit includes a locally developed structured checklist to ensure that the adolescent is adequately informed about the management of hypo- and hyperglycaemia, to confirm access to necessary diabetes-related equipment (e.g., Continuous Glucose Monitoring,CGM and insulin pump) and prescriptions, and to verify completion of relevant medical evaluations, including routine laboratory tests and blood pressure measurements. Adolescents transferred within the same hospital are offered group-based transfers, with some information jointly delivered by pediatric and adult diabetes teams. However, a small number of patients are transferred to adult care in the same hospital, and few of them attend this group session.

**Fig. 1:**
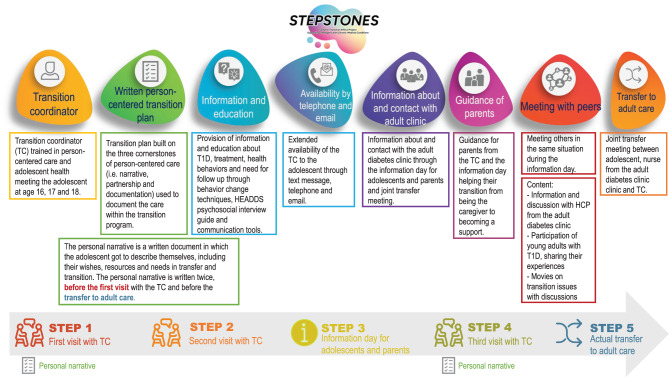
Outline of the STEPSTONES transition program

The STEPSTONES transition program is a complex intervention consisting of a generic, person-centered, structured transition program including eight components: a transition coordinator (TC); tailored education and discussions based on the adolescent’s needs; accessibility via phone, text, and email; information about and contact with the adult clinic; parental guidance; peer meetings; a person-centred transition plan; and preparation for the final transfer. These components are delivered in five steps: the first and second outpatient visits with the TC (at the age of 16 and 17 years); an information day for adolescents and parents; a third outpatient visit (at the age of 18 years); and, lastly, the transfer to adult care. The program is performed by a transition coordinator (TC, a specialized nurse at the outpatient pediatric clinic) (Fig. 1). At the time of this study, the RCT STEPSTONES-DIAB [17], conducted at the same clinics, was in its final phase. Participants in the control group received usual care. During the RCT, healthcare professionals received general information about the content of the intervention, but no in-depth information about the specific content of the intervention and how it was implemented. Therefore, health care professionals at the two clinics participating in this study were familiar with the content and structure of the program but had no deep insight and did not participate in providing the program as TCs.

### Participants

We adopted two types of purposeful sampling strategies [25], striving for maximum variation in relevant professional roles to achieve the aim of the study.

For registered nurses, we performed a total population sampling where we invited all registered nurses (*n* = 8) working at both outpatient diabetes clinics [26]. The nurse who had previously served as a TC in the RCT [17] was excluded from participating as an interviewee but took part in the interviews as a facilitator and observer. For physicians, we applied purposive strategic sampling [26] where physicians from both sites were strategically selected by the physician responsible for the diabetes team to achieve as much variation as possible To gain a societal and organizational perspective on barriers and facilitators, we invited the chair from the patient organization *Ung Diabetes (Young Diabetes)* and two healthcare managers to participate in the study.

### Data collection

Three face‒to-face focus group interviews were carried out, including three physicians and eight nurses (two with nurses and one with physicians). Individual interviews were conducted with the representative from the patient organization and the two healthcare managers (digitally via Microsoft Teams™). The focus group interviews were moderated by MS, with ALB as an observer, both registered nurses and with experience in transitional care and qualitative methodology. The individual interviews were conducted by the same researchers. The development of the interview guide was grounded in our recently published study [21] and was subsequently refined based on insights derived from that work, as well as the specific clinical context of the present study (Appendix 1). The interviews were conducted between 5 September and 15 October 2024. The focus groups lasted between 50 and 83 minutes, and the individual interviews lasted between 40 and 59 minutes. The interviews were audio recorded via an external recording device and transcribed verbatim.

### Data analysis

The data were analyzed using reflexive thematic analysis, following the approach outlined by Braun and Clarke [27]. All analyses were performed in Nvivo version 14 by two of the authors (ALB and CSL). The authors maintained an ongoing reflexive and transparent dialogue throughout the analytic process, and the preliminary final analysis was reviewed by MS as complementary perspective. The analysis was conducted in the following phases: 1) Familiarization with the data and 2) coding, where segments of data expressing barriers and facilitators for the implementation of the STEPSTONES transition program in usual care were captured. Coding discrepancies were not treated as errors but as opportunities to deepen analytic understanding. 3) Generated codes were grouped into potential themes based on patterns of barriers and facilitators. 4) The themes were systematically developed and reviewed to ensure that they accurately captured the underlying patterns of facilitators and barriers and that they were coherent and meaningful in the context of the data. 5) Through ongoing reflexive dialogue, themes were agreed upon based on their coherence, richness, and relevance to the research question, leading to the refinement and naming of emerging themes. 6) In the writing phase, the final themes were described and supported with illustrative quotes from the interviews.

## Results

In this study all registered nurses (*n* = 8) working at both outpatient diabetes clinics were interviewed and three physicians from both sites. The clinics have approximately ten physicians involved in diabetes care to varying degrees. Several were asked to participate but were unable to participate due to clinical circumstances. Further, one representative from the patient organization *Ung Diabetes (Young Diabetes)* and two healthcare managers were interviewed. Table 1 shows the background characteristics of the participants. Experience in pediatric diabetes care among participants ranged from 1 year to 28 years.

**Table 1:** Demographic characteristics of participants expressed in absolute numbers

	Nurses (n = 8)	Physicians (n = 3)	Managers (n = 2)	Patient organization (n = 1)	Total (n = 14)
Sex					
Female	7	3	1	1	12
Male	1	0	1	0	2
Site					
Site 1	5	2	*	N/A	7
Site 2	3	1	*	N/A	4

*Managers for both sites

Table 2 presents the overall theme, themes, and subthemes generated in the analysis. The barriers and facilitators are presented integrated within the results of the thematic analysis.

**Table 2:** Overview of the overall theme, themes (in bold) and subthemes (in italics)

**Overall theme:** Conceptualizations of structured transitional care		
Openness to changes in the transition care processes	Ensuring dedicated responsibility for transitional care	Supporting a youth-oriented approach
*An identified need for change*	*Clarifying roles is essential*	*Communicating with young people*
*Changes require time and commitment at all levels*	*Organizing transition-focused visits and forum*	*Fostering confidential relationships and partnerships with young people*
*Conditions for integrated care* remain a challenge	*Emphasis on structured interventions*	

### Openness to changes in the transition care processes

#### An identified need for change

In general, the HCPs, managers, and a patient representative described the need to prepare adolescents prior to the transition from pediatric to adult care. They agreed that this preparation should be initiated earlier than in current practice and needs to be extended over a longer period. Coordinated and structured implementation of the transition program was considered essential to ensure that young individuals receive the support they need during the transition. The participants emphasized the importance of having a youth-friendly attitude, noting that this approach is beneficial for patients’ health and well-being in the long term.

*It’s something that many of our members complain about, or they feel it’s a difficult period in life, and that … well, it simply needs to get better – Patient organization representative*

The HCPs emphasized the strong support within the team for changes in transitional care that may lead to improvements for patients and found it difficult to identify any barriers to implementation. A structured approach, such as the STEPSTONES program, was considered better than the current unstructured preparation. This could reduce the risk of important elements being overlooked, for example, when having a new HCP at the final visit, which is not uncommon at a large university hospital, due to high staff turnover.

*There are many elements that need to be structured and implemented over time so that it is no longer essentially just the final visit and maybe a bit of talking. Right now, it’s mostly loose conversation that doesn’t contribute very much – Physician 1*

#### Changes require time and commitment at all levels

The nurses stressed that the manager’s commitment and clear communication are vital for implementing a transition program. They considered managers’ attitudes to influence the allocation of time and resources, which was considered essential for success. Respecting the TCs’ time for transitional care and ensure that this is not subordinate to other duties. A shared understanding within the diabetes team was seen as crucial.

*I think it’s actually an advantage that there aren’t that many of us and that we have good communication. I still feel like that’s kind of the foundation for implementing- Nurse 1*

Managers emphasized the importance of being well informed about the transition program and adopting a solution-focused, holistic approach within their responsibilities. Ensuring adequate resource allocation and supporting key personnel were seen as essential for successful implementation. A facilitator was that a positive attitude toward structured transition programs was perceived among managers. A barrier was raised by a manager who emphasized the need to advocate for the program beyond the diabetes team, engaging both internal and external stakeholders as described by the head nurse down below. While new initiatives may invite skepticism, it was considered crucial that such doubt does not affect those directly involved in implementation.

*I must act as an advocate toward other groups. Many things are sometimes questioned, and my role is to keep that skepticism away from those who are doing the work. I also see it as an important role to explain things to people who don’t understand – Head nurse*

To facilitate successful implementation, managers stressed the importance of inclusive engagement, ensuring that no one feels excluded. Prioritizing individuals with a positive attitude was seen as beneficial, whereas acknowledging that others may need more time to embrace change. Both managers and HCPs emphasized the value of patience, recognizing that challenges are part of the process. Change takes time, and diverse responses are natural. The involvement of the entire diabetes team and fostering of mutual support throughout the process were considered essential.

As a barrier, some physicians viewed the program as overly ambitious, raising concerns that such ambition might hinder actual implementation. Nurses echo this concern, referencing past experiences where planned changes remained at the discussion stage without being realized.

*Certain things come up in team meetings month after month: ‘We should do this.’ Oh yes, we truly should! However, it just needs to actually be established: now we do this, it’s part of our routines, and it becomes clear to everyone – Nurse 1*

#### Conditions for integrated care remain a challenge

Improved collaboration between pediatric and adult diabetes clinics was emphasized by all respondents as a prerequisite for implementation. Managers and HCPs noted recent efforts to improve collaboration, including adult care clinics providing information to pediatric teams and organizing adult clinics. According to the patient organization representative, their members expressed a desire for coordinated transfer at the final visit. However, there was also an understanding that, from a logistical standpoint, this is not always feasible.

*We have always imagined having some kind of joint meeting -.with staff from both the pediatric and adult sides. That at least during the final visit in pediatric care, you would also get to meet the adult team, so you know who you will meet again in, say, three months when it’s time for the next appointment – Patient organization representative*

While participants expressed a desire for coordinated transition, logistical challenges such as the large number of adult care units and geographical distance between pediatric and adult care clinics were seen as barriers to implementing the program. Scheduling suitable times for all parties may also be challenging, especially given adolescents’ competing priorities and their legal autonomy at age 18, when they can choose whether to engage in healthcare services. One manager emphasized the need for sufficient resources in adult care to support young adults in a more efficient way.

*Part of it is about equipping adult healthcare, ensuring that these individuals are individuals who may be legally adults but still need support to manage their condition. You can’t expect them to go from 0 to 100% responsibility for making sure that follow-ups happen. So having that kind of focus, perhaps between ages 18 and 25 in adult units, or in youth units if they exist, is probably quite important – Head physician*

The patient representative described a disconnection between pediatric and adult care, with little to no contact between clinics prior to transition. They considered pediatric services to be reluctant to transfer patients due to concerns about reduced care quality in adult services. However, it has also been acknowledged that adult care generally involves more limited resources. One manager emphasized the importance of dialog between pediatric and adult care services to foster mutual understanding of the conditions and limitations within adult care.

### Ensuring dedicated responsibility for transitional care

#### Clarifying roles is essential

Ensuring that all staff are informed and understand their roles within the transition program was considered an essential facilitator. One manager also stressed the importance of assigning the right person to the right role for successful implementation. It was emphasized that TCs should possess substantial knowledge and specialization within the field.

*I think the transition coordinator is extremely important, someone who has more knowledge in adolescent medicine and in the entire transition process. That’s truly important because we do have quite a bit of staff turnover – Head nurse*

The appointment of a dedicated TC was perceived by most HCPs and the patient representative as a facilitating factor. This role ensures the structured preparation of adolescents for transition by systematically identifying those approaching the transition period and scheduling transitional care visits. Furthermore, according to HCPs, a designated TC could mitigate the risk of loss to follow-up by promoting continuity of care.

Collaboration between the TC and the patient’s primary nurse was considered essential. While views varied on whether the same person should manage both regular and transition visits, most agreed that the TC role is a distinct assignment influenced by the structure of a previous RCT conducted at the clinic. Coordinating the first transition visit with a regular nurse was suggested as beneficial. While long-term relationships with caregivers offer advantages, meeting a new professional may encourage openness around sensitive topics such as sexual health and substance use.

#### Organizing transition-focused visits and forums

An important facilitating factor was that managers and HCPs reported feeling well informed about the program and its scope, largely due to their knowledge of the prior RCT. They emphasized the importance of providing adolescents and their parents with timely information about the consultations within the transition program, distinguishing them from standard consultations and clearly communicating with the program’s objective. Ensuring that the content is engaging and relevant is seen as key to encouraging participation. One of the managers underlined the benefits of offering TC consultations as separate sessions, in addition to regular consultations, where discussions about standard medical concerns in diabetes (e.g., glucose levels) were intentionally avoided.

*I think it’s an enormous advantage to frame things clearly for the young person who now it’s okay to bring up these issues. This visit is not just about looking at glucose curves; now you can ask these questions – Head physician*

Nurses suggested that aligning TC consultations with routine appointments may improve attendance, which could be seen as a facilitator. Integrating transition discussions into standard nursing consultations was also suggested. Additionally, HCPs expressed concerns about how adolescents with complex needs, such as severe autism or intellectual disabilities, would be able to take part in the program.

One manager noted that youth group activities have been initiated over the years and are now viewed as a positive component of the transition program. Although these activities are not for all adolescents, for those who do participate, who are often encouraged by their parents, such group activities can be valuable.

#### Emphasis on structured interventions

The existing routine practices to promote transitional care differed between the units, with one having a structure and the other not. Based on experiences of implementing these transitional care structures, some barriers for the implementation of the Stepstones transition program were raised, such as the adolescent and parent information day, where previous endeavors had low attendance and limited interactions. Moreover, several respondents emphasized the importance of initiating the transition process earlier to better support adolescents.

Over the years, attempts have been made to encourage adolescents to reflect in advance on specific topics they wish to discuss during consultations, such as substance use and sexual health. Supporting documents have been developed, but these forms have rarely been completed and are no longer in use. As such, these prior experiences of failed implementation were considered potential barriers.

Nevertheless, the HCPs stressed the importance of having a structured approach, which they appreciated in the transition program. They highlighted that the structure ensures consistency and equity in care, reducing the risk of important elements being overlooked. The nurses highlighted the need to develop a structured approach for managing the waiting list system through which patients are scheduled for TC appointments and structured documentation of TC visits.

*It still feels like it might be easier to get things right and make these things happen when you have this structure, compared with the usual way of doing things. - Nurse 2*

*And then it becomes a source of reassurance … well, if you take STEPSTONES, you know how to do things, there is a structure – Nurse 3*

The nurses emphasized the value of having a facilitator to support consistent implementation, thereby reducing risk of any components of the program from being omitted and minimizing the risk of variation between sites. In addition to sharing experiences of transitional care, inspiration was also gained from meeting colleagues outside their own clinic who were further involved in the implementation process.

One of the managers underscored that there were generally positive attitudes toward improving the structure of transitional care, particularly within a person-centered framework. To meet the diverse needs of adolescents, it is essential to utilize the competencies of multidisciplinary teams effectively. Each professional may offer a unique perspective, and not every visit needs to address the same topics; varied approaches across consultations can provide more comprehensive support.

### Supporting a youth-oriented approach

#### Communicating with young people

The patient representative emphasized the importance of the program’s youth-friendly access to healthcare, such as the ability to send messages via a chat function at any time. As this was already implemented at both hospitals, it was seen as a facilitator for the transition program. As such, nurses identified potential benefits in expanding digital consultations, suggesting that reduced travel requirements may improve attendance among young patients. However, one manager noted persistent challenges with some technical systems.

*However, now I actually think it’s easier with the system, because they can communicate with us themselves without the parents being able to see it, for example. I think that’s convenient, or rather that it’s accessible – Nurse 4*

Several nurses raised the need for more training and education in adolescent medicine and how to communicate with adolescents to enable professional and effective execution of the TC role. One nurse expressed uncertainty about how to discuss sensitive topics such as substance use and sexual health with adolescents, as well as concerns about how to structure the final consultation before transfer to adult care. In the long term, it was considered desirable that this competence be provided more to nurses, with the aim of strengthening shared expertise and quality of care.

*The advantage is that the person who is the transition coordinator gains specific competence in transitional care and will likely learn over time what needs to be done and how to respond to different individuals - Head nurses*

#### Fostering confidential relationships and partnerships with young people

Several HCPs underlined the importance of having separate individual meetings with the adolescent and without the parents as pivotal during their teenage years, a core aspect of the STEPSTONES transition program. While some had already adopted this practice, there was a recognized need to initiate it earlier. Parental resistance to this aspect of the program was seen as a potential barrier. A potential barrier to adolescent-friendly care was the childlike design of some consultation rooms, thus suggesting the creation of environments that are more suitable and appealing to adolescents.

HCPs stressed the need for individualized transition processes, recognizing that patients’ needs vary despite having the same diagnosis. One nurse noted that diabetes care often remains family-centered and suggested that the transition program, being person-centered, could support a shift toward the approach to care.

One barrier expressed by HCPs was concerns that adolescents may not attend scheduled consultations within the transition program. They perceived from prior experience that adolescents already had the highest rates of cancellations and nonattendance, thus highlighting risks in implementation.

## Discussion

### Summary of the main findings

This study explored perceived barriers to and facilitators of the implementation of the STEPSTONES transition program for adolescents with T1D prior to implementation. Overall, the findings indicate a strong perceived need for structured transitional care, high professional motivation, and broad managerial support. Key facilitators included the program’s person-centered and structured design, alignment with existing (albeit unstructured) practices, and the appointment of a dedicated TC. Major barriers related to contextual constraints, such as limited resources, challenges in collaboration between pediatric and adult care, and healthcare professionals’ uncertainty regarding adolescent-focused communication and psychosocial support, are in line with findings from other contexts in Sweden [21].

Interpreted through the Integrated Promoting Action on Research Implementation in Health Services (i-PARIHS) framework [22], these findings illustrate that while the innovation itself (i.e., STEPSTONES transition program) is largely perceived as acceptable and relevant, successful implementation depends on recipients’ competence and confidence, contextual readiness across organizational boundaries, and the presence of active and sustained facilitation. Below, the findings are interpreted and discussed through the i-PARIHS framework.

### Innovation

Within the i-PARIHS framework, innovation characteristics such as clarity, evidence base, and compatibility with existing practices are central to implementation success [22]. The STEPSTONES transition program was perceived as a credible and meaningful innovation, largely because many of its components already exist in current practice in an informal or fragmented manner. This perceived familiarity reduced resistance to change and supports previous research suggesting that interventions perceived as “making visible what is already done” are more readily adopted in clinical settings [19].

The structured nature of STEPSTONES was consistently valued, echoing earlier findings from pediatric cardiology settings, where structure and defined components were associated with improved implementation fidelity [21]. However, concerns regarding the program’s ambition and scope were also raised. Similar concerns have been reported in other studies, where comprehensive programs risk being perceived as unrealistic in resource-constrained environments [28, 29]. From the i-PARIHS perspective, this underscores the importance of balancing intervention fidelity with local adaptability [22].

### Recipients

The users of innovations (i.e., recipients’) believe that skills and motivation are key determinants of implementation outcomes [22]. In this study, healthcare professionals expressed strong motivation to improve transitional care, aligning with prior research and guidelines showing high professional awareness of the transition gap in diabetes care [3]. Nurses viewed the role of TCs as meaningful and professionally affirming and as a possibility for continued professional development.

Moreover, uncertainty regarding communication with adolescents about sensitive psychosocial issues has emerged as a potential barrier. This finding is consistent with previous literature indicating that healthcare professionals often feel insufficiently prepared to address adolescent developmental and psychosocial needs, despite their recognition of their importance [21, 30]. Within i-PARIHS, such uncertainty reflects limited recipient readiness and highlights the need for targeted competence development to support the implementation of these core components of transitional care [22]. Moreover, the HCPs perceived adolescents as having the highest rates of cancellations and missed appointments, which raised concerns about the feasibility of implementation. These perceptions point to the need to consider how the program may require adaptation to better accommodate adolescents’ circumstances and engagement patterns, rather than assuming that existing structures of care are sufficient for this group.

### Context

Contextual factors (i.e., both inner and outer context relevant to the implementation) [22] were central in shaping perceptions of implementation feasibility. Leadership engagement and managerial support were repeatedly described as essential facilitators, particularly in securing protected time and legitimizing new roles within the team. This aligns with the literature identifying leadership as a critical contextual enabler [19, 22]. The need to clarify a new role, such as the TC, was emphasized by the participants in this study. In an umbrella review, the health care transition team has the following four basic responsibilities: (i) to educate patients, (ii) to provide age-appropriate care, (iii) to ensure continuity of care, and (iv) to coordinate the transition, and specialist nurses seem ideally placed to take the lead in the coordination of the transition [31].

However, broader system-level barriers were also prominent. Challenges in coordinating care across pediatric and adult services, the geographical dispersion of adult clinics, and limited adult care resources were perceived as significant obstacles. Similar cross-boundary challenges have been described in the literature, where discontinuities between pediatric and adult care undermine transitional practices [32, 33]. These findings highlight how macro- and meso-level contexts may constrain implementation despite favorable local conditions.

### Facilitation

Facilitation is positioned in i-PARIHS as the active mechanism that aligns innovation, recipients, and context [22]. Although not always explicitly labeled as such, the participants described clear needs for facilitative support, including guidance, follow-up, and cross-site learning. Nurses emphasized the importance of having access to a facilitator to ensure consistency and reducing risk of variation between clinics. A lack of explicit facilitation strategies represents potential vulnerability from an implementation perspective. Previous STEPSTONES research in cardiology demonstrated that structured facilitation within a randomized controlled trial contributed to high implementation fidelity [18]. Taken together, these findings suggest that deliberate facilitation, with a combination of internal facilitators with external or cross-organizational support, may be essential to translate readiness into sustained practice change [34].

Other perceived facilitating strategies to develop transitional care were related to specific components within Stepstones’ transition program. The participants in this study appreciated the emphasis on a youth-oriented approach, where tools such as HEADSS (Home Education Activities Depression Sexuality Substances - a psychosocial interview guide aimed at adolescents) may serve as support for adopting a more youth-centered practice, which is in line with international recommendations [3, 35]. In this way, practical tools contribute to shaping practices in accordance with established guidelines. In addition, having structured tools such as HEADSS may increase implementers capability in performing novel behavior and therefore lead to higher motivation to behavior change [36].

### Limitations

This study has several limitations that should be considered when interpreting the findings. First, the data were collected in two pediatric diabetes clinics within a single university hospital, which may limit transferability to other settings, particularly smaller clinics or healthcare systems with different organizational structures. Second, the participants were familiar with the STEPSTONES program through prior research involvement, which may have contributed to generally positive attitudes and underrepresentation of resistance. It is possible that this can be explained by loyalty to the program, on the other hand, the participants in this study had not been involved in providing the transition program, as a designated nurse was responsible for it within the framework of the RCT.

Huo et al. (2024) found that a supportive organizational climate for evidence-based practice was significantly and positively associated with nurses’ evidence-based practice behavior, suggesting that more evidence-oriented settings are more likely to implement evidence-based practices. Thus, process evaluations embedded within future implementation efforts are pivotal to capturing more nuanced impressions of implementation barriers and facilitators [37].

Third, the study explored perceptions prior to implementation; thus, reported facilitators and barriers reflect anticipated rather than experienced challenges. As such, findings should be interpreted as indicators of implementation readiness rather than predictors of actual implementation outcomes. Nevertheless, exploring perceptions at this stage is consistent with implementation science recommendations and provides valuable insights for proactive planning [38]. Finally, the low sample size, particularly in the physician group, warrants attention and should be interpreted with caution.

## Conclusion

This study demonstrates favorable preconditions for implementing the STEPSTONES transition program in pediatric diabetes care, characterized by strong professional motivation, perceived relevance of the innovation, and supportive leadership. However, successful implementation will depend on addressing contextual constraints across the pediatric–adult care interface, strengthening healthcare professionals’ competence in adolescent-centered communication, and investing in structured facilitation.

Interpreted post-hoc through the i-PARIHS framework, the findings emphasize that implementation success is unlikely to be achieved by focusing on the intervention alone. Instead, sustained facilitation that actively aligns innovation, recipients, and context will be critical. Future research should examine how these anticipated facilitators and barriers unfold during real-world implementation and explore facilitation strategies that support long-term integration of transitional care into routine diabetes services.

## Electronic supplementary material

Below is the link to the electronic supplementary material.

Supplementary Material 1

## Data Availability

The datasets analyzed in the current study are available from the corresponding author upon reasonable request.

## Abbreviations

CHD: Congenital Heart Disease
HCPs: Health care professionals
i-PARIHS: Integrated Promoting Action on Research Implementation in Health Services
MRC: Medical Research Council’s
TC: Transition Coordinator
T1D: Type 1 Diabetes
STEPSTONES: Swedish Transition Effects Project Supporting Teenagers with ChrONic mEdical conditions
RCT: Randomized Controlled Trial
SRQR: Standards for Reporting Qualitative Studies
