# Supplementary material for: Securing structured transitional care for adolescents with type 1 diabetes: a qualitative study of implementation barriers and facilitators prior to implementation– the STEPSTONES-Implement project
Source: BMC Health Serv Res. 2026 Apr 17;26:551. doi: 10.1186/s12913-026-14576-1 (PMC13094195; doi:10.1186/s12913-026-14576-1)
Supplement: Supplementary file 1 — Supplementary Material 1 [file 12913_2026_14576_MOESM1_ESM.pdf]

Appendix 1

Interview guide for interviews with healthcare staff and managers in outpatient care for adolescents with type 1 diabetes prior to the implementation of the STEPSTONES transition program

Overall purpose: To describe healthcare personnel's and managers' perceptions of the prerequisites and barriers for implementing Stepstones' transition program for young people with congenital heart disease.

Interview Guide – Interviews with Healthcare Personnel at Clinical Units

1. Can you describe what care looks like at your unit for adolescents transitioning to adult healthcare?
2. (Description of the components of the transition program) Based on this description, what are your thoughts on the transition program?
3. Which parts of the program do you think might be the most difficult to implement?
4. In what ways would the program need to be adapted to meet your needs?
5. As a nurse/physician, how do you view your role in the program?
6. As a nurse/physician, how do you view the collaboration with other professionals within the framework of the transition program?
7. Which factors at your workplace do you believe facilitate/ inhibit the implementation of the transition program?
8. What positive benefits could result from implementing the program at your clinic?
9. Which organizational factors do you believe influence the implementation of the program?
10. What support would you need during the implementation of the program?
11. If there were a person designated to support and coach the implementation of the program, who would this person be and what qualities would that person need to have?
12. Those were all the questions I had. Is there anything else you would like to add?

Interview Guide – Interviews with Managers and Decision-Makers

1. Can you describe what care looks like at your unit for adolescents transitioning to adult healthcare?
2. (Description of the components of the transition program) Based on this description, what potential barriers/ facilitators do you see for implementing the transition program at your clinic?
3. Which parts of the program do you think might be the most difficult to implement?
4. As a manager, how do you view your role in the program?
5. Which factors at your workplace do you believe facilitate/ inhibit the implementation of the transition program?

6. How do you view the willingness for change within your organization?
7. Which organizational factors do you believe influence the implementation of the program?
8. What support would your unit need during the implementation of the program?
9. If there were a person designated to support and coach the implementation of the program, who would this person be and what qualities would that person need to have?
10. Those were all the questions I had. Is there anything else you would like to add?
